# Cyclic-Dependent Damage Evolution in Self-Healing Woven SiC/[Si-B-C] Ceramic-Matrix Composites at Elevated Temperatures

**DOI:** 10.3390/ma13061478

**Published:** 2020-03-24

**Authors:** Longbiao Li, Pascal Reynaud, Gilbert Fantozzi

**Affiliations:** 1 College of Civil Aviation, Nanjing University of Aeronautics and Astronautics, No.29, Yudao St., Nanjing 210016, China; 2 Université de Lyon, INSA-Lyon, MATEIS (UMR CNRS 5510), 7 Avenue Jean Capelle, 69621 Villeurbanne Cedex, France; pascal.reynaud@insa-lyon.fr (P.R.); gilbert.fantozzi@insa-lyon.fr (G.F.)

**Keywords:** ceramic-matrix composites (CMCs), Self-healing, cycle-dependent, damage evolution, damage parameters, internal friction, interface damage, fiber failure

## Abstract

Cycle-dependent damage evolution in self-healing, 2.5D woven Hi-Nicalon^TM^ SiC/[Si-B-C] and 2D woven Hi-Nicalon^TM^ SiC/[SiC-B_4_C] ceramic-matrix composites (CMCs) at 600 and 1200 °C was investigated. The cycle-dependent damage parameters of internal friction, dissipated energy, Kachanov’s damage parameter, and broken fiber fraction were obtained to describe damage development in self-healing CMCs. The relationships between cycle-dependent damage parameters and multiple fatigue damage mechanisms were established. The experimental fatigue damage development of self-healing Hi-Nicalon^TM^ SiC/[Si-B-C] and Hi-Nicalon^TM^ SiC/[SiC-B_4_C] composites was predicted for different temperatures, peak stresses, and loading frequencies. The cycle-dependent damage evolution of self-healing Hi-Nicalon^TM^ SiC/[Si-B-C] and Hi-Nicalon^TM^ SiC/[SiC-B_4_C] composites depends on temperature, testing environment, peak stress, and loading frequency. For the Hi-Nicalon^TM^ SiC/[Si-B-C] composite, temperature is a governing parameter for the fatigue process. At an elevated temperature of 600 °C in an air atmosphere, the internal frictional parameter of Hi-Nicalon^TM^ SiC/[Si-B-C] composite decreases first and then increases with applied cycle number; however, at an elevated temperature of 1200 °C in an air atmosphere, the internal frictional parameter of Hi-Nicalon^TM^ SiC/[Si-B-C] composite decreases with applied cycle number, and the interface shear stress at 1200 °C is much lower than that at 600 °C. For Hi-Nicalon^TM^ SiC/[SiC-B_4_C] composite at 1200 °C, loading frequency is a governing parameter for the fatigue process. The degradation rate of interface shear stress is much higher at the loading frequency of 0.1 Hz than that at the loading frequency of 1 Hz.

## 1. Introduction

At present, superalloy is still the main material of any high temperature structure in an aeroengine (i.e., combustion chamber and turbine). After more than 40 years of development, the temperature resistance of metal materials represented by single crystal alloy has been greatly improved, but the difference between them and the combustion temperature of an aeroengine is still large, and the gap is gradually increasing in the new generation of aeroengines. In order to improve the temperature resistance, most designers adopt the active cooling of “thermal barrier coating + film cooling.” However, the introduction of cooling air affects the combustion efficiency, and the higher the combustion temperature, the greater the effect. Therefore, the improvement of temperature resistance is the key technology for developing next generation aeroengines. Ceramic materials with high temperature resistance, good mechanical properties, and low density have long been considered ideal materials for high-temperature structures in aeroengines. However, due to the low toughness of ceramics, once damaged, they will cause catastrophic consequences for aeroengine, which limits their application. In order to improve the toughness of ceramic materials, fiber-reinforced ceramic-matrix composites (CMCs) have been developed. The SiC_f_/SiC composite possesses low density and a long lifetime at high temperatures for up to thousands of hours, making it an ideal material for the hot section components of commercial aeroengines [1,2,3,4]. Under long-term applications at elevated temperatures, CMCs are subjected to mechanical or thermal cyclic fatigue loading [5,6,7]. Understanding the fatigue damage mechanisms of CMCs at elevated temperatures is necessary for hot section components designers [8,9,10]. To reduce the failure risk of CMC hot section components in aeroengines during operation, it is necessary to investigate the cycle-dependent fatigue damage evolution in a high temperature environment, and to develop related damage models, prediction methods, and computation tools [11,12,13].

Oxidation is the key factor to limiting the application of CMCs on hot section load-carrying components of aeroengines. Combining carbides deposited by the chemical vapor infiltration (CVI) process with specific sequences, a new generation of SiC/SiC composite with a self-healing matrix has been developed to improve the oxidation resistance [14,15]. The self-sealing matrix forms a glass with oxygen at high temperature and consequently prevents oxygen diffusion inside the material. At low temperature of 650–1000 °C in dry and wet oxygen atmospheres, the self-healing 2.5D Nicalon^TM^ NL202 SiC/[Si-B-C] with a pyrocarbon (PyC) interphase exhibits a better oxidation resistance compared to SiC/SiC with PyC, due to the presence of boron compounds [16]. The fatigue lifetime duration in an air atmosphere at intermediate and high temperatures is considerably reduced beyond the elastic yield point. For the Nicalon^TM^ SiC/[Si-B-C] composite, the elastic yield point is about σ = 80 MPa. The lifetime duration is about t = 10–20 h at T = 873 K and less than t = 1 h at T = 1123 K under σ_max_ = 120 MPa. For the self-healing Hi-Nicalon^TM^ SiC/SiC composite, a duration of t = 1000 h without failure is reached at σ_max_ = 170 MPa, and a duration higher than t = 100 h at σ_max_ = 200 MPa and T = 873 K is reached [17]. For the self-healing Hi-Nicalon^TM^ SiC/[SiC-B_4_C] composite, at 1200 °C, there is little influence on the fatigue performance at f = 1.0 Hz, but there is a noticeably degraded fatigue lifetime at f = 0.1 Hz with the presence of steam [18,19]. Increase in temperature from T = 1200 to 1300 °C slightly degrades the fatigue performance in an air atmosphere, but not in a steam atmosphere [20]. The crack growth in the SiC fiber controls the fatigue lifetime of self-healing Hi-Nicalon^TM^ SiC/[Si-B-C] at T = 873 K; and the fiber creep controls the fatigue lifetime of self-healing SiC/[Si-B-C] at T = 1200 °C [21]. The typical cyclic fatigue behavior of a self-healing Hi-Nicalon^TM^ SiC/[Si-B-C] composite involves an initial decrease of the effective modulus to a minimum value, followed by a stiffening, and the time-to-the minimum modulus is in inverse proportion to the loading frequency [22]. The initial cracks within the longitudinal tows caused by interphase oxidation contribute to the initial decrease of modulus. The glass produced by the oxidation of the self-healing matrix may contribute to the stiffening of the composite either by sealing the cracks or by bonding the fiber to the matrix [23]. The damage evolution of self-healing Hi-Nicalon^TM^ SiC/[Si-B-C] composite at elevated temperature can be monitored using acoustic emission (AE) [24,25]. The relationship between interface oxidation and AE energy under static fatigue loading at elevated temperatures has been developed [26]. However, at a high temperature above 1000 °C, AE cannot be applied for cyclic fatigue damage monitoring. The complex fatigue damage mechanisms of self-healing CMCs affect damage evolution and lifetime. Hysteresis loops are related to cycle-dependent fatigue damage mechanisms [27,28,29]. The damage parameters derived from hysteresis loops have already been applied for analyzing the fatigue damage and fracturing of different non-oxide CMCs at elevated temperatures [30,31,32,33]. However, the cycle-dependent damage evolution and accumulation of self-healing CMCs have very different values from previous analysis results, especially at elevated temperatures. In the research mentioned above, the relationship between the cycle-dependent damage evolution and mechanical hysteresis of self-healing CMCs has not been established.

The objective of this paper is to analyze cycle-dependent damage development in self-healing 2.5D woven Hi-Nicalon^TM^ SiC/[Si-B-C] and 2D woven Hi-Nicalon^TM^ SiC/[SiC-B_4_C] composites at T = 600 °C and 1200 °C using damage evolution models and parameters. The cycle-dependent damage parameters of internal friction, dissipated energy, Kachanov’s damage parameter, and broken fiber fraction were obtained to analyze damage development in self-healing CMCs. The relationships between cycle-dependent damage parameters and multiple fatigue damage mechanisms are established. The experimental fatigue damage evolution of each of the composites—self-healing Hi-Nicalon^TM^ SiC/[Si-B-C] and Hi-Nicalon^TM^ SiC/[SiC-B_4_C]—is predicted. The effects of fatigue peak stress, testing environment, and loading frequencies on the evolution of internal damage and final fracture are analyzed.

## 2. Materials and Experimental Procedures

The Hi-Nicalon^TM^ fibers (Nippon Carbon Co., Ltd., Tokyo, Japan) with an interphase of pyrolytic carbon (PyC) reinforced multilayered matrix [Si-B-C] were provided by SNECMA Propulsion Solide, Le Haillan, France. The self-healing Hi-Nicalon^TM^ SiC/[Si-B-C] composite was fabricated using chemical vapor infiltration (CVI). The experimental results were performed and obtained by Penas [34]. The detailed information of materials and experimental procedures of 2.5D Hi-Nicalon^TM^ SiC/[Si-B-C] composite at 600 °C and 1200 °C in an air atmosphere are shown in Table 1. The 2.5D fiber preform was consolidated by an interphase PyC deposited by CVI with a thickness of 0.1 μm to optimize the interphase properties and promote the desired pseudo-ductile behavior in the composite. The fiber volume was about 35%, and the porosity was about 10%.

The fatigue specimen was dog bone-shaped, and the dimensions were 200 mm total length, 5 mm thickness, and 16 mm width in the gage section. The fatigue experiments at elevated temperature were conducted in an INSTRON Model 8502 (INSTRON System Corp., Norwood, MA, USA) and an AET furnace system (AET Technologies Corp., Meylan, France). The fatigue tests were on a sinusoidal wave form and the loading frequency was f = 0.25 Hz. For tension–compression fatigue tests, the minimum stress is always equal to σ_min_ = −50 MPa; and for tension–tension fatigue tests, the minimum stress is set to be σ_min_ = 0 MPa, and the fatigue limit was defined to be N = 1,000,000.

The Hi-Nicalon^TM^ SiC/[SiC-B_4_C] composite was reinforced with Hi-Nicalon^TM^ fibers with the PyC interphase (about 0.4 μm thick) and boron carbide overlay (about 1.0 μm thick), and was processed via CVI by Hyper-Therm High-Temperature Composites, Inc. (Huntington Beach, CA, USA). The experimental results were obtained by Ruggles-Wrenn et al. [18]. The detailed information of materials and experimental procedures is shown in Table 2. The fiber volume was about 34.8%, and the composite density was about 2.56 g/cm^3^. The dimensions of the dog bone-shaped specimen were 152 mm total length, 10 mm width, and 3.5 mm thickness in the gage section. The fatigue tests were performed in an MTS 810 (MTS, Eden Prairie, MN, USA). The fatigue tests were performed under load control with a stress ratio of R = 0.05 and f = 0.1 and 1.0 Hz. The fatigue peak stress was σ_max_ = 140 MPa. The fatigue limit was N = 100,000 at f = 0.1 Hz, and N = 200,000 at f = 1.0 Hz.

## 3. Theoretical Analysis

**Figure 1:**
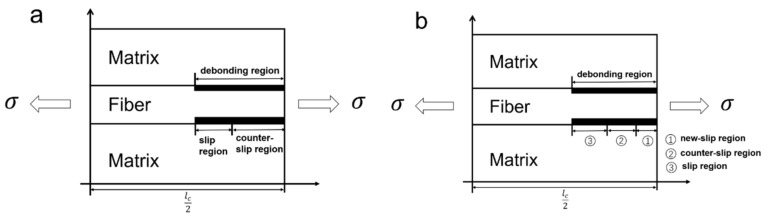
Unit cell of damaged CMCs upon (**a**) unloading and (**b**) reloading.

When the peak stress is higher than the first matrix cracking stress, under cyclic fatigue loading, multiple fatigue damage mechanisms of matrix cracking, interface debonding, wear and oxidation, and fiber fracturing occur [5,6,7,8,35,36]. Hysteresis loops appear and evolve with cycle number upon unloading and reloading due to internal multiple damages in CMCs [27,28,29,30,31,32,33,37]. A unit cell is extracted from the damaged CMCs, as shown in Figure 1. The total length of the unit cell is half of a matrix crack spacing (l_c_/2), and the interface debonding length between the space of matrix cracking is l_d_. Upon unloading, the debonding zone is divvied into a counter-slip zone with the length of l_y_ and a slip zone of length l_d_–l_y_, as shown in Figure 1a; and upon reloading, the debonding zone is divvied into a new slip zone with a length of l_z_, a counter-slip region with a length of l_y_–l_z_, and a slip region with a length of l_d_–l_y_, as shown in Figure 1b.

Based on the interface debonding and slip state between the matrix crack spacing, the types of hysteresis loops can be divided into four cases, as shown in Table 3.

For Case 1 and 2 in Table 3, the unloading and reloading composite hysteresis strain is a function of cycle-dependent unloading intact fiber stress (Φ_U_(N)), reloading intact fiber stress (Φ_R_(N)), interface shear stress (τ_i_(N)), interface debonding, and slip length (l_d_(N), l_y_(N), and l_z_(N)). The cycle-dependent unloading composite hysteresis strain (ε_U_(N)) and reloading composite hysteresis strain (ε_R_(N)) are determined as:

(1) $$\begin{array}{l} \varepsilon_{U}\left(N\right)=\frac{\Phi_{U}\left(N\right)+\varphi \left(N\right)}{E_{f}}+4\frac{\tau_{i}\left(N\right)}{E_{f}}\frac{l_{y}^{2}\left(N\right)}{r_{f}l_{c}}\\ -\frac{\tau_{i}\left(N\right)}{E_{f}}\frac{\left(2l_{y}\left(N\right)-l_{d}\left(N\right)\right)\left(2l_{y}\left(N\right)+l_{d}\left(N\right)-l_{c}\left(N\right)\right)}{r_{f}l_{c}}-\left(\alpha_{c}-\alpha_{f}\right)\Delta Τ \end{array}$$

(2) $$\begin{array}{l} \varepsilon_{R}\left(N\right)=\frac{\Phi_{R}\left(N\right)+\varphi \left(N\right)}{E_{f}}-4\frac{\tau_{i}\left(N\right)}{E_{f}}\frac{l_{z}^{2}\left(N\right)}{r_{f}l_{c}}+4\frac{\tau_{i}\left(N\right)}{E_{f}}\frac{{\left(l_{y}\left(N\right)-2l_{z}\left(N\right)\right)}^{2}}{r_{f}l_{c}}\\ +2\frac{\tau_{i}\left(N\right)}{E_{f}}\frac{\left(l_{d}\left(N\right)-2l_{y}\left(N\right)+2l_{z}\left(N\right)\right)\left(l_{d}\left(N\right)+2l_{y}\left(N\right)-2l_{z}\left(N\right)-l_{c}\left(N\right)\right)}{r_{f}l_{c}}-\left(\alpha_{c}-\alpha_{f}\right)\Delta Τ \end{array}$$

where r_f_ is the fiber radius; α_f_ and α_c_ denote the fiber and composite’s thermal expansion coefficient; and ΔT denotes the temperature difference between tested and fabricated temperatures.

For Cases 3 and 4 in Table 3, the cycle-dependent unloading composite hysteresis strain (ε_U_(N)) and reloading composite hysteresis strain (ε_R_(N)) can be expressed as:

(3) $$\varepsilon_{U}\left(N\right)=\frac{\Phi_{U}\left(N\right)+\Delta \varphi \left(N\right)}{E_{f}}+4\frac{\tau_{i}\left(N\right)}{E_{f}}\frac{l_{y}^{2}\left(N\right)}{r_{f}l_{c}}-2\frac{\tau_{i}\left(N\right)}{E_{f}}\frac{{\left(2l_{y}\left(N\right)-l_{c}\left(N\right)/2\right)}^{2}}{r_{f}l_{c}}-\left(\alpha_{c}-\alpha_{f}\right)\Delta Τ$$

(4) $$\begin{array}{l} \varepsilon_{R}\left(N\right)=\frac{\Phi_{R}\left(N\right)+\Delta \varphi \left(N\right)}{E_{f}}-4\frac{\tau_{i}\left(N\right)}{E_{f}}\frac{l_{z}^{2}\left(N\right)}{r_{f}l_{c}}+4\frac{\tau_{i}\left(N\right)}{E_{f}}\frac{{\left(l_{y}\left(N\right)-2l_{z}\left(N\right)\right)}^{2}}{r_{f}l_{c}}\\ -2\frac{\tau_{i}\left(N\right)}{E_{f}}\frac{{\left(l_{c}/2-2l_{y}\left(N\right)+2l_{z}\left(N\right)\right)}^{2}}{r_{f}l_{c}}-\left(\alpha_{c}-\alpha_{f}\right)\Delta Τ \end{array}$$

The cycle-dependent internal damage parameter is defined by ∆W/W_e_, where W_e_ is the maximum elastic energy stored during a cycle [30].

(5) $$\Delta W\left(N\right)=\int_{\sigma_{\min}}^{\sigma_{\max}}\left[\varepsilon_{U}\left(N\right)-\varepsilon_{R}\left(N\right)\right]d\sigma$$

Substituting Equations (1)–(4) into Equation (5), the damage parameter (ΔW(N)) can be obtained, which is a function of cycle-dependent unloading intact fiber stress (Φ_U_(N)); reloading intact fiber stress (Φ_R_(N)); interface shear stress (τ_i_(N)); interface debonding; and slip length, (l_d_(N), l_y_(N), and l_z_(N)). It should be noted that the cycle-dependent unloading intact fiber stress (Φ_U_(N)) and reloading intact fiber stress (Φ_R_(N)) consider fiber failure and broken fiber fraction. Comparing experimental ∆W/W_e_ or ∆W with theoretical values, the interface shear stress (τ_i_(N)) and broken fiber fraction (P) can be obtained for different cycle numbers.

The mean elastic modulus (E) is the mean slope of hysteresis loop. This modulus is usually normalized by the Young’s modulus E_0_ of a undamaged composite, leading to the plotting of (E/E_0_). A Kachanov’s damage parameter (D) is given by:

(6) $$D=1-\frac{E}{E_{0}}$$

The Kachanov’s damage parameter (D) is another way to describe the evolution of a composite’s mean elastic modulus (E) under cyclic fatigue but contains the same information as the normalized modulus (E/E_0_).

## 4. Experimental Comparisons

The monotonic tensile and cycle-dependent damage evolutions of self-healing 2.5D woven Hi-Nicalon^TM^ SiC/[Si-B-C] and 2D woven Hi-Nicalon^TM^ SiC/[SiC-B_4_C] composites were analyzed for different temperatures. The monotonic tensile curves exhibit obvious nonlinearity at elevated temperatures. For 2.5D woven Hi-Nicalon^TM^ SiC/[Si-B-C] at 600 °C and 1200 °C, the tensile curves can be divided into three main zones; however, for 2D woven Hi-Nicalon^TM^ SiC/[SiC-B_4_C] at 1200 °C, the tensile curve can only be divided into two main zones. The cycle-dependent damage parameters of internal friction (∆W(N)/W_e_(N)), dissipated energy (∆W(N)), interface shear stress (τ_i_(N)), Kachanov’s damage parameter (D(N)), and broken fiber fraction (P(N)) versus cycle number are analyzed for different temperatures, peak stresses, and loading frequencies. The interface shear stress decreases with applied cycle number; and the Kachanov’s damage parameter and broken fiber fraction increase with applied cycle number. However, the evolution of internal frictional and dissipated energy with more cycles is much more complex, as together, they depend on the peak stress, temperature, and testing environment. The internal damage evolution of each composite—2.5D woven Hi-Nicalon^TM^ SiC/[Si-B-C] and 2D woven Hi-Nicalon^TM^ SiC/[SiC-B_4_C]—when subjected to cyclic fatigue loading, was obtained.

### 4.1. 2.5D Woven Hi-Nicalon^TM^ SiC/[Si-B-C] at 600 °C in an Air Atmosphere

**Figure 2:**
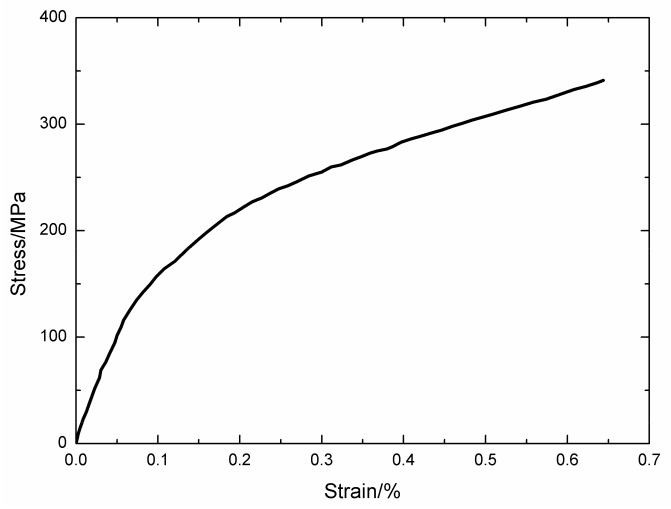
Tensile curve of 2.5D woven self-healing Hi-Nicalon^TM^ SiC/[Si-B-C] composite at 600 °C in an air atmosphere.

The monotonic tensile curve of 2.5D woven self-healing Hi-Nicalon^TM^ SiC/[Si-B-C] composite at T = 600 °C in an air atmosphere is shown in Figure 2. The self-healing Hi-Nicalon^TM^ SiC/[Si-B-C] composite fractures at σ_UTS_ = 341 MPa with the failure strain of ε_f_ = 0.64%. The tensile curve exhibits obvious nonlinearity, and can be divided into three zones, including: (1) the linear elastic zone with an elastic modulus of E_c_ = 195 ± 20 GPa; (2) the nonlinear zone due to multiple matrix cracking; and (3) the second linear zone after saturation of matrix cracking up to final fracture, with an elastic modulus of E_c_ = 23 ± 1 GPa, which is half of theoretical value E_f_V_fl_ (43 GPa) when the load is supported only by the longitudinal fiber.

**Figure 3:**
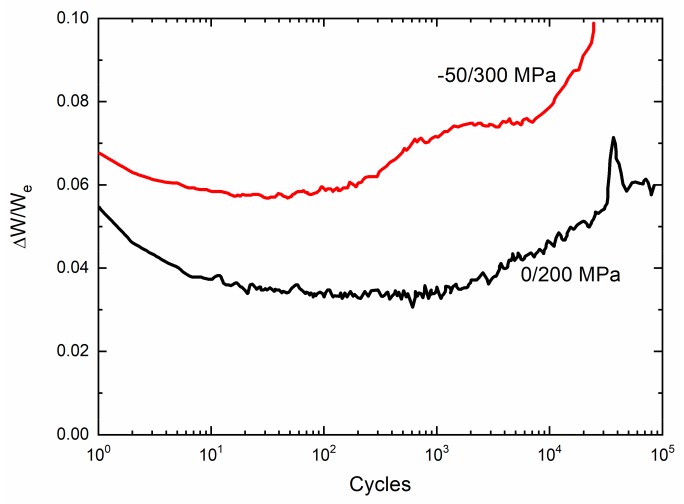
Experimental ∆W/W_e_ versus cycle number curves of 2.5D woven self-healing Hi-Nicalon^TM^ SiC/[Si-B-C] composite at 600 °C in an air atmosphere.

The experimental cycle-dependent internal friction parameter (∆W/W_e_) versus cycle number curves of 2.5D woven self-healing Hi-Nicalon^TM^ SiC/[Si-B-C] composite under σ_min_ = −50/σ_max_ = 300 MPa and σ_min_ = 0/σ_max_ = 200 MPa at T = 600 °C in an air atmosphere are shown in Figure 3. The cycle-dependent internal friction parameter (∆W/W_e_) decreases first, followed by a short stabilization, and increases again before reaching a plateau; and finally, there is a sharp increase when the composite approaches failure. During initial stage of cyclic fatigue loading, matrix cracking and interface debonding occur when the fatigue peak stress is higher than the first matrix cracking stress. Under repeated unloading and reloading, the sliding between the fiber and the matrix leads to the interface wear and oxidation, which decreases the interface shear stress [5,8,27,29]. The initial decrease of internal friction parameter (∆W/W_e_) is mainly attributed to matrix cracking, cycle-dependent interface debonding, and interface wear. However, with increasing cycle number, the interface wear and oxidation decrease the interface shear stress to a constant value [30], leading to the stabilization of cycle-dependent interface debonding and slip length and also the internal friction damage parameter (∆W/W_e_). The interface wear and oxidation also decrease the fiber strength, leading to the gradual fracture of fiber, and the sudden increase of internal friction at the end of the test corresponding to fiber broken [30,33].

**Figure 4:**
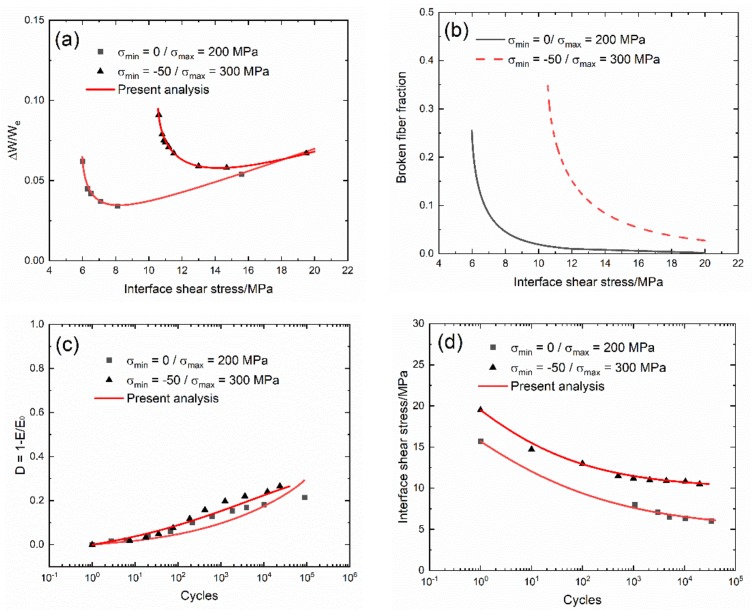
(**a**) Experimental and predicted internal friction parameter (ΔW/W_e_) versus the interface shear stress curves: (**b**) the broken fiber fraction (P) versus the interface shear stress curves; (**c**) the experimental and predicted Kachanov’s damage parameter (D) versus cycle number curves; and (**d**) the interface shear stress (τ_i_(N)) versus cycle number curve of 2.5D woven self-healing Hi-Nicalon^TM^ SiC/[Si-B-C] composite at 600 °C in an air atmosphere.

The experimental and predicted cycle-dependent internal friction parameter (∆W(N)/W_e_(N)) and broken fiber fraction (P(N)) versus the interface shear stress curves, and the cycle-dependent Kachanov’s damage parameter (D(N)) and the interface shear stress (τ_i_(N)) versus cycle number curves of 2.5D woven self-healing Hi-Nicalon^TM^ SiC/[Si-B-C] at 600 °C in an air atmosphere are shown in Figure 4 and Table 4. Under σ_max_ = 200 and 300 MPa, the internal damage parameter (∆W(N)/W_e_(N)) first decreases with the interface shear stress, mainly due to the interface wear and oxidation, and then increases with the interface shear stress, mainly due to the fiber broken, corresponding to the interface slip Case 4 in Table 3, as shown in Figure 4a. Under σ_max_ = 300 MPa, the broken fiber fraction (P(N)) at higher interface shear stress is much higher than that under σ_max_ = 200 MPa, mainly due to higher peak stress, as shown in Figure 4b; and the Kachanov’s damage parameter (D(N)) is also higher than that under σ_max_ = 200 MPa, as shown in Figure 4c. The interface shear stress under σ_max_ = 300 MPa is also higher than that under σ_max_ = 200 MPa, mainly due to the scatter of interface shear stress or compressive stress of σ_min_ = −50 MPa acting on the composite.

Under σ_max_ = 200 MPa, the cycle-dependent damage parameter (∆W/W_e_) decreases first, i.e., from ∆W/W_e_ = 0.054 at τ_i_ = 15.7 MPa to ∆W/W_e_ = 0.034 at τ_i_ = 8.0 MPa, and then increases from ∆W/W_e_ = 0.034 at τ_i_ = 8.0 MPa to ∆W/W_e_ = 0.062 at τ_i_ = 6.0 MPa. The cycle-dependent broken fiber fraction (P) increases from P = 0.004 at τ_i_ = 15.7 MPa to P = 0.24 at τ_i_ = 6.0 MPa. The cycle-dependent Kachanov’s damage parameter (D) increases from D = 0 at N = 1 to D = 0.21 at N = 89,459. The cycle-dependent interface shear stress (τ_i_) decreases from τ_i_ = 15.7 MPa at N = 1 to τ_i_ = 6.0 MPa at N = 33,788.

Under the peak stress of σ_max_ = 300 MPa, the cycle-dependent damage parameter (∆W/W_e_) decreases first, i.e., from ∆W/W_e_ = 0.067 at τ_i_ = 19.5 MPa to ∆W/W_e_ = 0.058 at τ_i_ = 14.7 MPa; and then increases from ∆W/W_e_ = 0.058 at τ_i_ = 14.7 MPa to ∆W/W_e_ = 0.091 at τ_i_ = 10.5 MPa. The cycle-dependent broken fiber fraction (P) increases from P = 0.029 at τ_i_ = 19.5 MPa to P = 0.347 at τ_i_ = 10.5 MPa. The cycle-dependent Kachanov’s damage parameter (D) increases from D = 0 at N = 1 to D = 0.265 at N = 23,666. The interface shear stress (τ_i_) decreases from τ_i_ = 19.5 MPa at N = 1 to τ_i_ = 10.5 MPa at N = 19,812.

### 4.2. 2.5D Woven Hi-Nicalon^TM^ SiC/[Si-B-C] at 1200 °C in an Air Atmosphere

**Figure 5:**
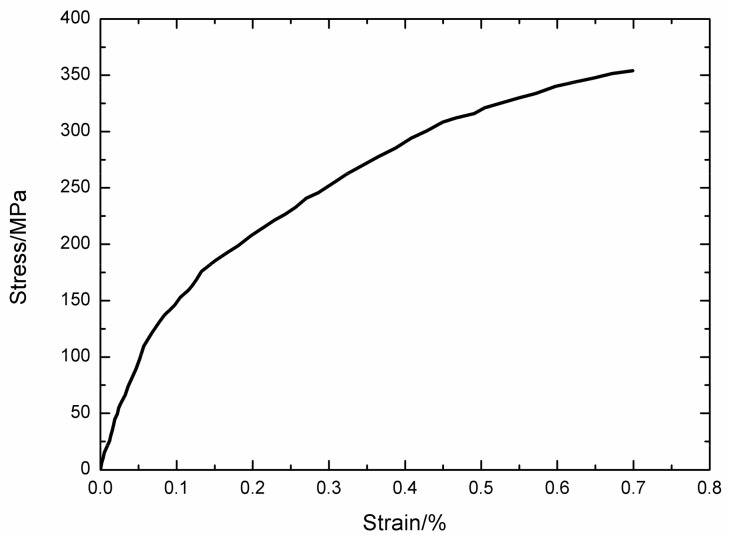
Tensile curve of 2.5D woven self-healing Hi-Nicalon^TM^ SiC/[Si-B-C] composite at 1200 °C in an air atmosphere.

The monotonic tensile curve of 2.5D woven self-healing Hi-Nicalon^TM^ SiC/[Si-B-C] composite at T = 1200 °C in an air atmosphere is shown in Figure 5. The composite tensile fractured at σ_UTS_ = 354 MPa with ε_f_ = 0.699%. The tensile curve can also be divided into three zones, including: (1) the initial linear elastic zone; (2) the non-linear zone; and (3) the second linear region with fiber broken. The average fracture strength and failure strain of 2.5D woven Hi-Nicalon^TM^ SiC/[Si-B-C] composite are slightly lower at T = 1200 °C; i.e., σ_UTS_ = 320 MPa and ɛ_f_ = 0.62%, against σ_UTS_ = 332 MPa and ɛ_f_ = 0.658% at T = 600 °C.

**Figure 6:**
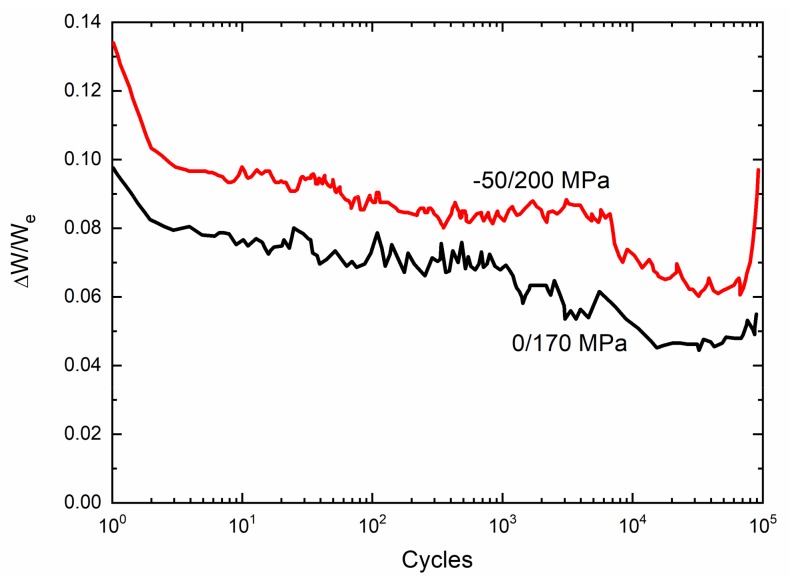
Experimental internal friction parameter (∆W/W_e_) versus cycle number curves of 2.5D woven self-healing Hi-Nicalon^TM^ SiC/[Si-B-C] composite at 1200 °C in an air atmosphere.

The experimental cycle-dependent internal friction parameter (∆W/W_e_) versus cycle number curves of 2.5D woven self-healing Hi-Nicalon^TM^ SiC/[Si-B-C] at T = 1200 °C in an air atmosphere are shown in Figure 6. The cycle-dependent internal friction parameter (∆W/W_e_) decreases continuously, and finally, there is a sharp increase when the composite approaches failure. The internal friction decreases as the interface wear reduces the interface shear stress. The sudden increase of internal friction at the end of the test corresponds to the fiber broken.

**Figure 7:**
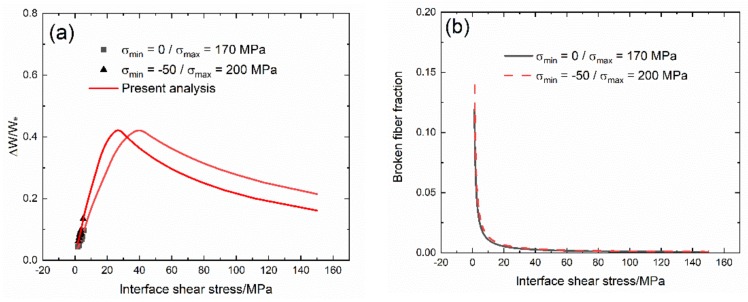

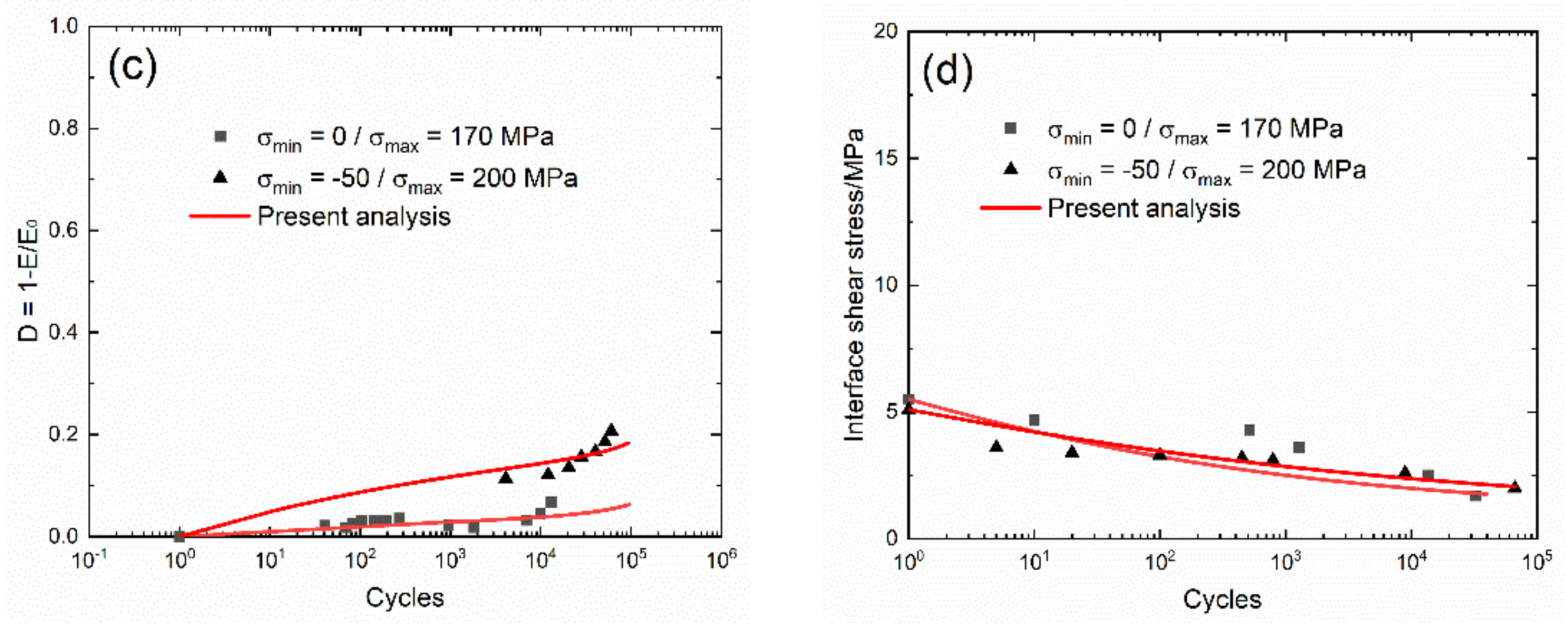
(**a**) Experimental and predicted internal friction parameter (ΔW/W_e_) versus the interface shear stress curves: (**b**) the broken fiber fraction (P) versus the interface shear stress curves; (**c**) the experimental and predicted Kachanov’s damage parameter (D) versus cycle number curves; and (**d**) the interface shear stress versus cycle number curves of 2.5D woven self-healing Hi-Nicalon^TM^ SiC/[Si-B-C] composite at 1200 °C in an air atmosphere.

The experimental and predicted cycle-dependent internal friction parameter (∆W(N)/W_e_(N)) and the broken fiber fraction (P(N)) versus the interface shear stress curves; and the cycle-dependent Kachanov’s damage parameter (D(N)) and the interface shear stress (τ_i_(N)) versus cycle number curves of 2.5D woven self-healing Hi-Nicalon^TM^ SiC/[Si-B-C] composite at 1200 °C in an air atmosphere are shown in Figure 7 and Table 5. Under σ_max_ = 170 and 200 MPa, the internal damage parameter (∆W/W_e_) decreases with the interface shear stress, corresponding to the interface slip case (Case 4) in Table 3. Under σ_max_ = 200 MPa, the broken fiber fraction (P) is higher than that under σ_max_ = 170 MPa at the same interface shear stress; and the Kachanov’s damage parameter (D) is also higher than that under σ_max_ = 170 MPa at the same cycle number. However, the value of the interface shear stress under σ_max_ = 200 MPa is close to that under σ_max_ = 170 MPa.

Under σ_max_ = 170 MPa, the cycle-dependent internal friction parameter (∆W/W_e_) increases to the peak value first and then decreases; i.e., from ∆W/W_e_ = 0.2 at τ_i_ = 150 MPa to ∆W/W_e_ = 0.42 at τ_i_ = 39.5 MPa, and then to ∆W/W_e_ = 0.04 at τ_i_ = 1.25 MPa. The broken fiber fraction (P) increases from P = 0.0007 at τ_i_ = 150 MPa to P = 0.12 at τ_i_ = 1.2 MPa. The Kachanov’s damage parameter (D) increases from D = 0 at N = 1 to D = 0.068 at N = 13,202. The interface shear stress (τ_i_) decreases from τ_i_ = 5.5 MPa at N = 1 to τ_i_ = 1.7 MPa at N = 32334.

Under σ_max_ = 200 MPa, the cycle-dependent internal friction parameter (∆W/W_e_) increases to the peak value first and then decreases; i.e., from ∆W/W_e_ = 0.161 at τ_i_ = 150 MPa to ∆W/W_e_ = 0.42 at τ_i_ = 26.4 MPa, and then to ∆W/W_e_ = 0.05 at τ_i_ = 1.37 MPa. The broken fiber fraction (P) increases from P = 0.0008 at τ_i_ = 150 MPa to P = 0.14 at τ_i_ = 1.3 MPa. The Kachanov’s damage parameter (D) increases from D = 0 at N = 1 to D = 0.2 at N = 60530. The interface shear stress (τ_i_) decreases from τ_i_ = 5.1 MPa at N = 1 to τ_i_ = 2 MPa at N = 66794.

### 4.3. 2D Woven Self-healing Hi-Nicalon^TM^ SiC/[SiC-B_4_C] at 1200 °C in Air and in a Steam Atmospheres

**Figure 8:**
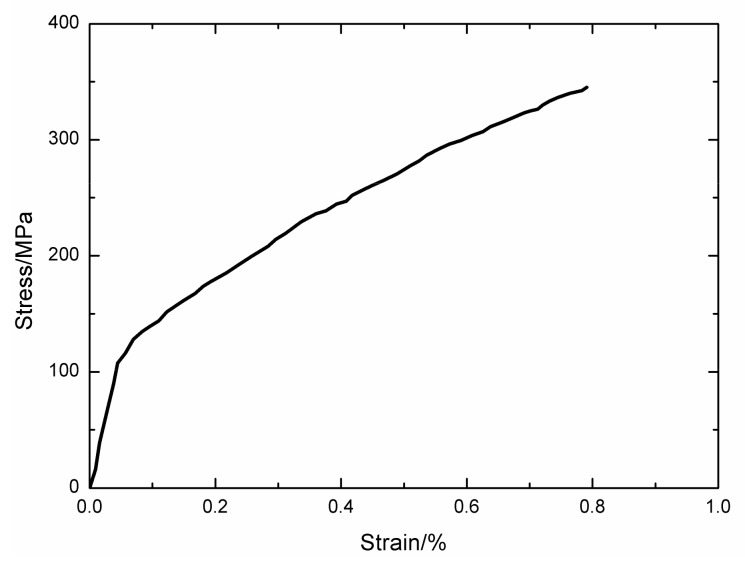
Tensile curve of 2D woven self-healing Hi-Nicalon^TM^ SiC/[SiC-B_4_C] composite at 1200 °C.

**Figure 9:**
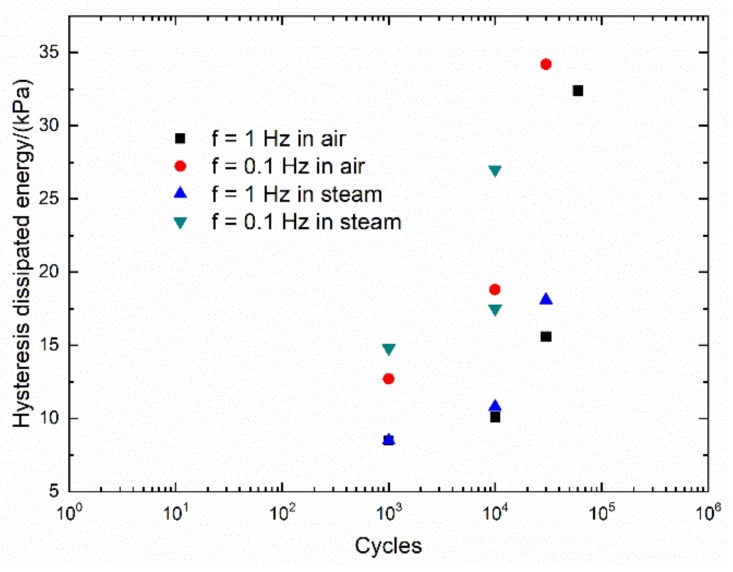
Experimental cycle-dependent dissipated energy (ΔW) versus cycle number curves of 2D woven self-healing Hi-Nicalon^TM^ SiC/[SiC-B_4_C] composite under σ_max_ = 140 MPa at 1200 °C in air and in steam atmospheres.

The monotonic tensile curve of 2D woven self-healing Hi-Nicalon^TM^ SiC/[SiC-B_4_C] composite at T = 1200 °C in an air atmosphere is shown in Figure 8. The composite tensile fractured at σ_UTS_ = 345 MPa with ε_f_ = 0.79%. The experimental cycle-dependent dissipated energy (ΔW(N)) versus cycle number curves of 2D woven self-healing Hi-Nicalon^TM^ SiC/[SiC-B_4_C] composite under σ_max_ = 140 MPa at T = 1200 °C in an air atmosphere and in a steam atmosphere with f = 0.1 and 1 Hz are shown in Figure 9.

**Figure 10:**
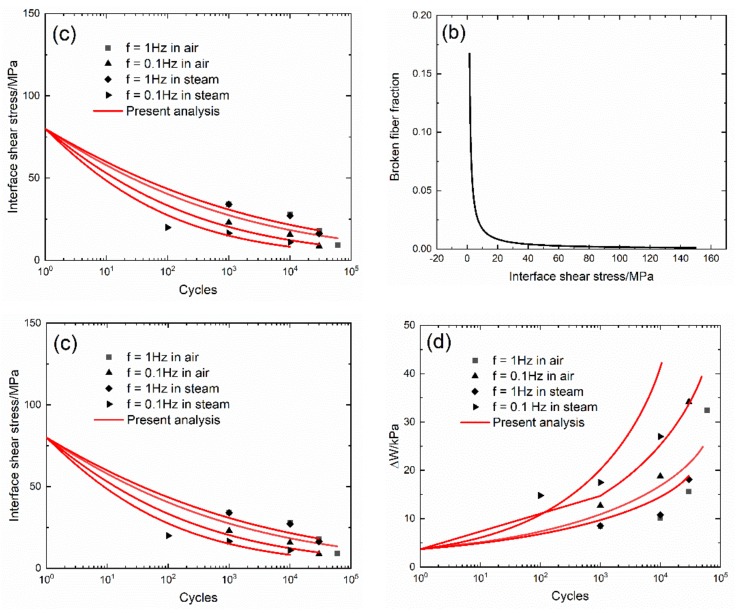
(**a**) Experimental and predicted dissipated energy (ΔW) versus the interface shear stress curves: (**b**) the broken fiber fraction (P) versus the interface shear stress curves; (**c**) the experimental and predicted interface shear stress (τ_i_) versus cycle number curves; and (**d**) the experimental and predicted dissipated energy (ΔW) versus cycle number curves of 2D woven self-healing Hi-Nicalon^TM^ SiC/[SiC-B_4_C] composite at 1200 °C in an air atmosphere.

The experimental and predicted cycle-dependent dissipated energy (ΔW(N)) and broken fiber fraction (P(N)) versus the interface shear stress curves, and the cycle-dependent interface shear stress (τ_i_(N)) and dissipated energy (ΔW(N)) versus cycle number curves of 2D woven self-healing Hi-Nicalon^TM^ SiC/[SiC-B_4_C] composite under σ_max_ = 140 MPa and f = 0.1 and 1 Hz at T = 1200 °C in air and in steam atmospheres are shown in Figure 10 and Table 6. The damage parameter (ΔW) increases with the interface shear stress, corresponding to the interface slip case (Case 2) in Table 3, as shown in Figure 10a. The broken fiber fraction (P) increases with decreasing interface shear stress, as shown in Figure 10b. The interface shear stress degrades with cycle number, and the degradation rate of the interface shear stress under f = 0.1 Hz is higher than that under f = 1 Hz. When f = 1 Hz, the degradation rate of the interface shear stress is slightly affected in air or in steam; however, when f = 0.1 Hz, the degradation rate of the interface shear stress in steam is higher than that in air.

At T = 1200 °C in air, under σ_max_ = 140 MPa and f = 1 Hz, the experimental cycle-dependent dissipated energy (ΔW) increases from ΔW = 8.5 kPa at τ_i_ = 34 MPa to ΔW = 32.4 kPa at τ_i_ = 9.2 MPa; the broken fiber fraction (P) increases from P = 0.001 at τ_i_ = 150 MPa to P = 0.167 at τ_i_ = 1.57 MPa; the experimental interface shear stress (τ_i_) decreases from τ_i_ = 34 MPa at N = 1000 to τ_i_ = 9.2 MPa at N = 60,000; and the experimental dissipated energy (ΔW) increases from ΔW = 8.4 kPa at N = 1000 to ΔW = 32.4 kPa at N = 60,000.

Under σ_max_ = 140 MPa and f = 0.1 Hz, the experimental cycle-dependent dissipated energy (ΔW) increases from ΔW = 12.7 kPa at τ_i_ = 23 MPa to ΔW = 34.2 kPa at τ_i_ = 8.7 MPa; the broken fiber fraction (P) increases from P = 0.001 at τ_i_ = 150 MPa to P = 0.167 at τ_i_ = 1.57 MPa; the experimental interface shear stress (τ_i_) decreases from τ_i_ = 23 MPa at N = 1000 to τ_i_ = 8.7 MPa at N = 30,000; and the experimental dissipated energy (ΔW) increases from ΔW = 12.7 kPa at N = 1000 to ΔW = 34.2 kPa at N = 30,000.

At T = 1200 °C in steam, under σ_max_ = 140 MPa and f = 1 Hz, the experimental cycle-dependent dissipated energy (ΔW) increases from ΔW = 8.5 kPa at τ_i_ = 34 MPa to ΔW = 18.1 kPa at τ_i_ = 16.3 MPa; the broken fiber fraction (P) increases from P = 0.001 at τ_i_ = 150 MPa to P = 0.167 at τ_i_ = 1.57 MPa; the experimental interface shear stress (τ_i_) decreases from τ_i_ = 34 MPa at N = 1000 to τ_i_ = 16.3 MPa at N = 30,000; and the experimental dissipated energy (ΔW) increases from ΔW = 8.5 kPa at N = 1000 to ΔW = 18.1 kPa at N = 30,000.

At T = 1200 °C in steam, under σ_max_ = 140 MPa and f = 0.1 Hz, the experimental cycle-dependent dissipated energy (ΔW) increases with decreasing interface shear stress—i.e., from ΔW = 14.8 kPa at τ_i_ = 20 MPa to ΔW = 27 kPa at τ_i_ = 11 MPa; the broken fiber fraction (P) increases from P = 0.001 at τ_i_ = 150 MPa to P = 0.167 at τ_i_ = 1.57 MPa; the experimental interface shear stress (τ_i_) decreases from τ_i_ = 20 MPa at N = 100 to τ_i_ = 11 MPa at N = 10000; and the experimental dissipated energy (ΔW) increases from ΔW = 14.8 kPa at N = 100 to ΔW = 27 kPa at N = 10000.

### 4.4. Discussion

**Figure 11:**
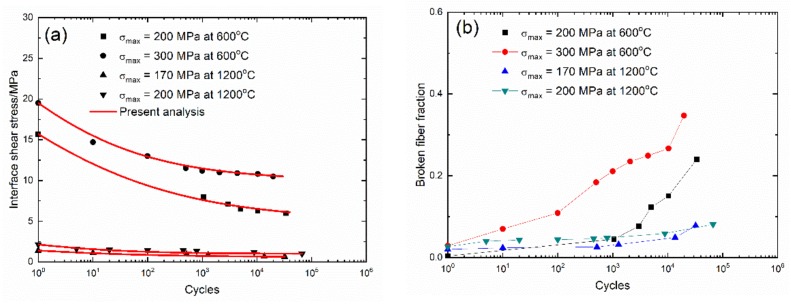
(**a**) Experimental and predicted interface shear stress (τ_i_) versus cycle number curves; and (**b**) the broken fiber fraction (P) versus cycle number curves of 2.5D woven self-healing Hi-Nicalon^TM^ SiC/[Si-B-C] composite at 600 °C and 1200 °C in air and in steam atmospheres.

The evolution curves of cycle-dependent interface shear stress (τ_i_(N)) and broken fiber fraction (P(N)) versus cycle number of 2.5D woven self-healing Hi-Nicalon^TM^ SiC/[Si-B-C] composite under different peak stresses at T = 600 °C and 1200 °C in an air atmosphere are shown in Figure 11. For 2.5D SiC/[Si-B-C] composite, the temperature is a governing parameter for the fatigue damage process. When the temperature increases from T = 600 °C to 1200 °C, the interface shear stress decreases a lot, and the interface shear stress degradation rate and broken fiber fraction increase with peak stress.

At T = 600 °C and σ_max_=200 MPa, the interface shear stress (τ_i_) decreases from τ_i_ = 15.7 MPa at N = 1 to τ_i_ = 6.0 MPa at N = 33788, and the broken fiber fraction (P) increases from P = 0.004 to P = 0.24; and under σ_max_ = 300 MPa, the interface shear stress (τ_i_) decreases from τ_i_ = 19.5 MPa at N = 1 to τ_i_ = 10.5 MPa at N = 19,812, and the broken fiber fraction (P) increases from P = 0.029 to P = 0.347. However, at T = 1200 °C and under σ_max_ = 170 MPa, the interface shear stress (τ_i_) decreases from τ_i_ = 5.5 MPa at N = 1 to τ_i_ = 1.7 MPa at N = 32,334, and the broken fiber fraction (P) increases from P = 0.0007 to P = 0.12; and under σ_max_ = 200 MPa, the interface shear stress (τ_i_) decreases from τ_i_ = 5.1 MPa at N = 1 to τ_i_ = 2 MPa at N = 66794, and the broken fiber fraction (P) increases from P = 0.0008 to P = 0.14.

**Figure 12:**
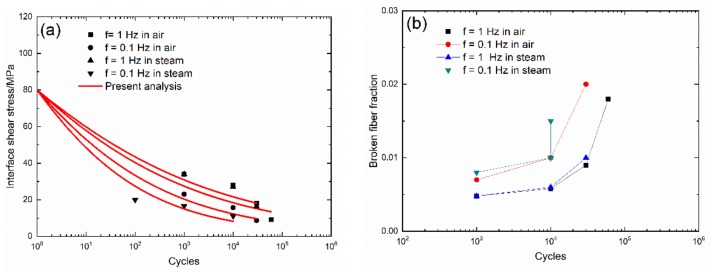
(**a**) Experimental and predicted interface shear stress (τ_i_) versus cycle number curves; and (**b**) the broken fiber fraction (P) versus cycle number curves of 2D woven self-healing Hi-Nicalon^TM^ SiC/[SiC-B_4_C] composite under σ_max_ = 140 MPa at 1200 °C in air and in steam atmospheres.

The evolution curves of cycle-dependent interface shear stress (τ_i_(N)) and the broken fiber fraction (P(N)) versus cycle number of 2D woven self-healing Hi-Nicalon^TM^ SiC/[SiC-B_4_C] composite under σ_max_ = 140 MPa at T = 1200 °C in air and in steam atmospheres with f = 0.1 and 1Hz are shown in Figure 12. For the 2D SiC/[SiC-B_4_C] composite, the loading frequency is the governing parameter for the fatigue process. Under σ_max_ = 140 MPa, at the same cycle number, the interface shear stress (τ_i_) decreases with decreasing loading frequency, and the interface shear stress in a steam atmosphere is lower than that in an air atmosphere; and the broken fiber fraction (P) increases with decreasing loading frequency, and the broken fiber fraction in a steam atmosphere is higher than that in an air atmosphere.

At T = 1200 °C in an air atmosphere with f = 1 Hz, the interface shear stress (τ_i_) decreases from τ_i_ = 34 MPa at N = 1000 to τ_i_ = 9.2 MPa at N = 60,000, and the broken fiber fraction (P) increases from P = 0.0048 to P = 0.018; when f = 0.1 Hz, the interface shear stress (τ_i_) decreases from τ_i_ = 23 MPa at N = 1000 to τ_i_ = 8.7 MPa at N = 30,000, and the broken fiber fraction (P) increases from P = 0.007 to P = 0.02. At T = 1200 °C in a steam atmosphere and f = 1 Hz, the interface shear stress (τ_i_) decreases from τ_i_ = 34 MPa at N = 1000 to τ_i_ = 16.3 MPa at N = 30,000, and the broken fiber fraction (P) increases from P = 0.0048 to P = 0.01; and when f = 0.1 Hz, the interface shear stress (τ_i_) decreases from τ_i_ = 20 MPa at N = 100 to τ_i_ = 11 MPa at N = 10000, and the broken fiber fraction (P) increases from P = 0.008 to P = 0.015.

## 5. Conclusions

In this paper, cycle-dependent damage evolution of self-healing 2.5D woven Hi-Nicalon^TM^ SiC/[Si-B-C] and 2D woven Hi-Nicalon^TM^ SiC/[SiC-B_4_C] composites under different peak stresses and loading frequencies at T = 600 °C and 1200 °C are investigated. The damage parameters of internal friction (∆W/W_e_), dissipated energy (∆W), Kachanov’s damage parameter (D), broken fiber fraction (P), and interface shear stress (τ_i_) are used to describe fatigue damage evolution.

(1) For 2.5D woven self-healing Hi-Nicalon^TM^ SiC/[Si-B-C] composite, temperature is a governing parameter for the fatigue process. At T = 600 °C in an air atmosphere, ∆W/W_e_ first decreases and then increases with cycle number; and at T = 1200 °C in an air atmosphere, ∆W/W_e_ decreases with cycle number. The degradation rate of the interface shear stress and broken fiber faction increases with peak stress.
(2) For 2D woven self-healing Hi-Nicalon^TM^ SiC/[SiC-B_4_C] composite at T = 1200 °C, loading frequency is a governing parameter for the fatigue process. ∆W increases with cycle number; under σ_max_ = 140 MPa, at the same applied cycle number, the interface shear stress decreases with the loading frequency, and the interface shear stress in a steam atmosphere is lower than that in an air atmosphere, and the broken fiber fraction increases with decreasing loading frequency, and the broken fiber fraction in a steam atmosphere is higher than that in an air atmosphere.

## Figures and Tables

**Table 1:** Materials and experimental procedures of 2.5D woven Hi-Nicalon^TM^ SiC/[Si-B-C] composite at 600 °C and 1200 °C in an air atmosphere.

Materials	
Fiber	Hi-Nicalon^TM^
Interphase	Pyrolytic Carbon (PyC)
Matrix	Multilayer [Si-B-C]
Fiber preform	2.5D
Fiber volume/(%)	35
Fabrication method	Chemical Vapor Infiltration (CVI)
Manufacturer	SNECMA Propulsion Solide
**Experimental Procedures**	
Specimen shape	Dog bone-shaped
Specimen dimension	200 mm length 5 mm thickness 16 mm width
Testing machine	INSTRON Model 8502 servo hydraulic load-frame
Loading frequency/(Hz)	0.25
Maximum cycle number	1000000
Temperature/(°C)	600, 1200
Environment	air
σ_min_/σ_max_ (MPa)	0/200, −50/300 at 600 °C 0/170, −50/200 at 1200 °C

**Table 2:** Materials and experimental procedures of 2D woven Hi-Nicalon^TM^ SiC/[SiC-B_4_C] composite at 1200 °C in air and steam atmospheres.

Materials	
**Fiber**	Hi-Nicalon^TM^
Interphase	Pyrolytic Carbon Boron carbide
Matrix	Multilayer [SiC-B_4_C]
Fiber preform	2D
Fiber volume/(%)	34.8
Fabrication method	Chemical Vapor Infiltration (CVI)
Manufacturer	Hyper-Therm High-Temperature Composites, Inc.
**Experimental Procedures**	
Specimen shape	Dog bone-shaped
Specimen dimension	152 mm length 3.5 mm thickness 10 mm wide
Testing machine	MTS 810 servo hydraulic load-frame
Loading frequency/(Hz)	0.1 and 1
Maximum cycle number	100,000 for f = 0.1 Hz 200,000 for f = 1 Hz
Temperature/(°C)	1200
Environment	air and steam
σ_min_/σ_max_ (MPa)	7/140

**Table 3:** Interface debonding and slip state in CMCs.

Case	Interface Debonding Condition	Interface Counter Slip Condition	Interface New Slip Condition
Case 1	l_d_(σ_max_) < l_c_/2	l_y_(σ_min_) = l_d_(σ_max_)	l_z_(σ_max_) = l_d_(σ_max_)
Case 2	l_d_(σ_max_) < l_c_/2	l_y_(σ_min_) < l_d_(σ_max_)	l_z_(σ_max_) < l_d_(σ_max_)
Case 3	l_d_(σ_max_) = l_c_/2	l_y_(σ_min_) < l_c_/2	l_z_(σ_max_) < l_c_/2
Case 4	l_d_(σ_max_) = l_c_/2	l_y_(σ_min_) = l_c_/2	l_z_(σ_max_) = l_c_/2

**Table 4:** Cycle-dependent damage evolution of 2.5D woven self-healing Hi-Nicalon^TM^ SiC/[Si-B-C] composite at 600 °C in an air atmosphere.

0/200 MPa at 600 °C in Air Atmosphere			
Cycle number	∆W/W_e_	τ_i_/MPa	P/%
1	0.054	15.7	0.4
1055	0.034	8.0	4.5
2993	0.037	7.1	7.7
5041	0.042	6.5	12.3
10,343	0.045	6.3	15.1
33,788	0.062	6	24.0
**−50/300 MPa at 600 °C in Air Atmosphere**			
Cycle number	∆W/W_e_	τ_i_/MPa	P/%
1	0.067	19.5	2.9
10	0.058	14.7	7.0
100	0.059	13.0	10.9
500	0.067	11.5	18.4
1000	0.071	11.2	21.1
2080	0.074	11.0	23.5
4410	0.075	10.9	24.9
10,406	0.079	10.8	26.7
19,812	0.091	10.5	34.7

**Table 5:** Cycle-dependent damage evolution of 2.5D woven self-healing Hi-Nicalon^TM^ SiC/[Si-B-C] composite at 1200 °C in an air atmosphere.

0/170 MPa at 1200 °C in Air Atmosphere			
Cycle number	∆W/W_e_	τ_i_/MPa	P/%
1	0.097	5.5	2.0
10	0.076	4.7	2.4
517	0.068	4.3	2.6
1281	0.062	3.6	3.2
13,700	0.047	2.5	4.9
32,334	0.044	1.7	7.8
**−50/200 MPa at 1200 °C in Air Atmosphere**			
Cycle number	∆W/W_e_	τ_i_/MPa	P/%
1	0.133	5.1	2.7
5	0.096	3.6	4.0
20	0.090	3.4	4.3
100	0.087	3.3	4.4
450	0.085	3.2	4.6
790	0.082	3.1	4.8
8910	0.073	2.6	5.9
66,794	0.061	2.0	8.1

**Table 6:** Cycle-dependent damage evolution of 2D woven self-healing Hi-Nicalon^TM^ SiC/[SiC-B_4_C] composite under *σ*_max_ = 140 MPa at 1200 °C in air and in steam atmospheres.

σ_max_ = 140 MPa and f = 1.0 Hz at 1200 °C in Air Atmosphere			
Cycle number	∆W/kPa	τ_i_/MPa	P/%
1000	8.5	34	0.48
10,000	10.1	28	0.58
30,000	15.6	18	0.9
60,000	32.4	9.2	1.8
**σ_max_ = 140 MPa and f = 0.1 Hz at 1200 °C in Air Atmosphere**			
Cycle number	∆W/kPa	τ_i_/MPa	P/%
1000	12.7	23	0.7
10,000	18.8	15.7	1
30,000	34.2	8.7	2
**σ_max_ = 140 MPa and f = 1.0 Hz at 1200 °C in Steam Atmosphere**			
Cycle number	∆W/kPa	τ_i_/MPa	P/%
1000	8.5	34	0.48
10,000	10.8	27.1	0.6
30,000	18.1	16.3	1
**σ_max_ = 140 MPa and f = 0.1 Hz at 1200 °C in Steam Atmosphere**			
Cycle number	∆W/kPa	τ_i_/MPa	P/%
1000	14.8	20	0.8
10,000	17.5	16.5	1
10,000	27	11	1.5

## Nomenclature

*r*_f_	fiber radius
*l*_c_	matrix crack spacing
*l*_d_	fiber/matrix interface debonding length
*l*_y_	fiber/matrix interface counter slip length
*l*_z_	fiber/matrix interface new slip length
*σ*_max_	fatigue peak stress
*σ*_min_	fatigue valley stress
Φ_U_	intact fiber stress upon unloading
Φ_R_	intact fiber stress upon reloading
ε_U_	unloading composite strain
ε_R_	reloading composite strain
τ_i_	fiber/matrix interface shear stress
α_f_	fiber thermal expansional coefficient
α_c_	composite thermal expansional coefficient
ΔT	temperature difference between tested and fabricated temperature
*E*_f_	fiber elastic modulus
*W*_e_	maximum elastic energy stored during the cycle
ΔW	hysteresis dissipated energy
*E*_0_	Young’s modulus of undamaged composite
*E*	mean slope of hysteresis loop
*D*	Kachanov’s damage parameter
